# *Glaesserella parasuis* QseBC two-component system senses epinephrine and regulates *capD* expression

**DOI:** 10.1128/spectrum.01508-23

**Published:** 2023-10-26

**Authors:** Ju Sun, Siting Wen, Zhichao Wang, Wei Liu, Yan Lin, Jiayun Gu, Weiting Mao, Xiaojuan Xu, Qigai He, Xuwang Cai

**Affiliations:** 1 National Key Laboratory of Agricultural Microbiology, College of Veterinary Medicine, Huazhong Agricultural University, Wuhan, Hubei, China; 2 Key Laboratory of Preventive Veterinary Medicine in Hubei Province, Cooperative Innovation Center for Sustainable Pig Production, Huazhong Agricultural University, Wuhan, Hubei, China; Institut Pasteur, Paris, France

**Keywords:** *Glaesserella parasuis*, capsular polysaccharide, epinephrine, two-component system

## Abstract

*Glaesserella parasuis*, a common inhabitant of the upper respiratory tract of pigs, is the etiological agent of Glässer’s disease, which is characterized by fibrinous polyserositis, polyarthritis, and meningitis. Decreased production of capsular polysaccharide (CPS) promotes bacterial adhesion and uptake, whereas maximal expression of CPS is essential to evade the host immune response. For bacterial survival under highly variable host conditions, a coordinated regulation of CPS expression is necessary. In the present study, we investigated how epinephrine (Epi) affects the CPS production, biofilm formation, and adhesion ability of *G. parasuis*, as well as the specific mechanism of *G. parasuis* changes in response to Epi. The results demonstrated that Epi stimulation dramatically inhibited CPS expression, and both the action of Epi and the deletion of CPS biosynthesis gene *capD* had detrimental impacts on biofilm formation, and had the enhanced ability for cell adhesion. QseBC two-component system is a typical adrenaline-sensing system in bacteria. In the present study, by quantitative real-time PCR, Western blot, and electrophoretic mobility shift assay, we discovered that in the presence of Epi, *G. parasuis* negatively regulated *capD* expression *via* the interaction of QseB with the promoter of *capD*, thereby affecting the synthesis of CPS. In conclusion, we explored the impact of Epi on CPS synthesis and defined its regulatory mechanism in *G. parasuis* for the first time, which provides an important theoretical basis for understanding the pathogenic mechanism of *G. parasuis*.

**IMPORTANCE:**

The key bacterial pathogen *Glaesserella parasuis*, which can cause Glässer’s disease, has caused significant financial losses to the swine industry worldwide. Capsular polysaccharide (CPS) is an important virulence factor for bacteria, providing the ability to avoid recognition and killing by the host immune system. Exploring the alteration of CPS synthesis in *G. parasuis* in response to epinephrine stimulation can lay the groundwork for revealing the pathogenic mechanism of *G. parasuis* as well as providing ideas for Glässer’s disease control.

## INTRODUCTION

*Glaesserella parasuis*, formerly known as *Haemophilus parasuis*, is the causative agent of Glässer’s disease characterized by fibrinous polyserositis, polyarthritis, and meningitis (1). When breeding conditions suddenly change, *G. parasuis* can infect the pigs with low resistibility (2). In addition, *G. parasuis* is frequently co-infected with porcine reproductive and respiratory syndrome virus. It induces intense inflammatory reactions and accelerates the death of the piglets, leading to enormous financial losses for the global pig industry (3 – 5).

Production of an extracellular layer of polysaccharide, termed the capsule polysaccharide, is a common feature of numerous bacteria (6). During infection, the amount of capsular polysaccharide (CPS) is a double-edged sword for bacteria. A reduction in the amount of capsular material might significantly enhance adherence and uptake (7), whereas it may also convert the bacteria to become stronger in terms of their ability to elude the host immune system (8). As a result, for a pathogen to colonize, survive, and disseminate within the host, both the conversion from highly encapsulated to less encapsulated bacteria and the retrograde conversion must be carefully regulated (9). However, the mechanism through which bacteria regulate the production of CPS remains largely unknown. The *capD* gene, which encodes a polysaccharide biosynthesis protein, has been identified as a novel pathogenicity-associated determinant of *G. parasuis* (10). *G. parasuis* isolates with *capD* are highly and moderately virulent (11, 12), and CapD expression is upregulated *in vitro* (13). Therefore, understanding the transcriptional mechanisms of *capD* will help to explain the molecular mechanisms of adhesion, colonization, and invasion of *G. parasuis*.

Two-component systems (TCS) are the dominant form of genetic control in response to environmental stimuli in bacteria (14). In a broad spectrum of bacterial species, the QseBC TCS as a quorum-sensing system is conserved (15). The QseBC controls the expression of some genes linked to metabolism, virulence, and stress response (16). In *Edwardsiella tarda* and enterohemorrhagic *Escherichia coli*, the QseBC regulates the transcription of the genes encoding the flagellar proteins in an epinephrine (Epi)- and norepinephrine-dependent manner to control bacterial motility (17, 18). In the presence of signal molecules, QseC works as sensor and receives signals, and then transfers the phosphate to the intracellular response regulator QseB. Therefore, QseC inhibitors have been developed as novel antimicrobials (19), and QseC may also be utilized as a potential subunit vaccine candidate (20). In addition, the activation pattern of QseC and the regulatory effects of QseB on virulence genes differ between bacteria (21). The investigation of the relationship between QseBC and the virulence gene *capD* will help to explain the pathogenic mechanism of *G. parasuis*.

In the present study, we demonstrated that the response regulator QseB is positively controlled by itself in the current of Epi, which is related to the regulation of CPS synthesis gene *capD*. As a result, CPS synthesis was inhibited, leading to increased adhesion and reduced biofilm formation of *G. parasuis*. These results will help us better understand the colonization and pathogenic mechanism of *G. parasuis*. Reducing stress in pigs is necessary for the prevention and treatment of Glässer’s disease, and the development of TCS inhibitors may also be more meaningful in disease control.

## RESULTS

### Epi affects CPS synthesis, adhesion and biofilm formation in *G. parasuis*

**Fig 1:**
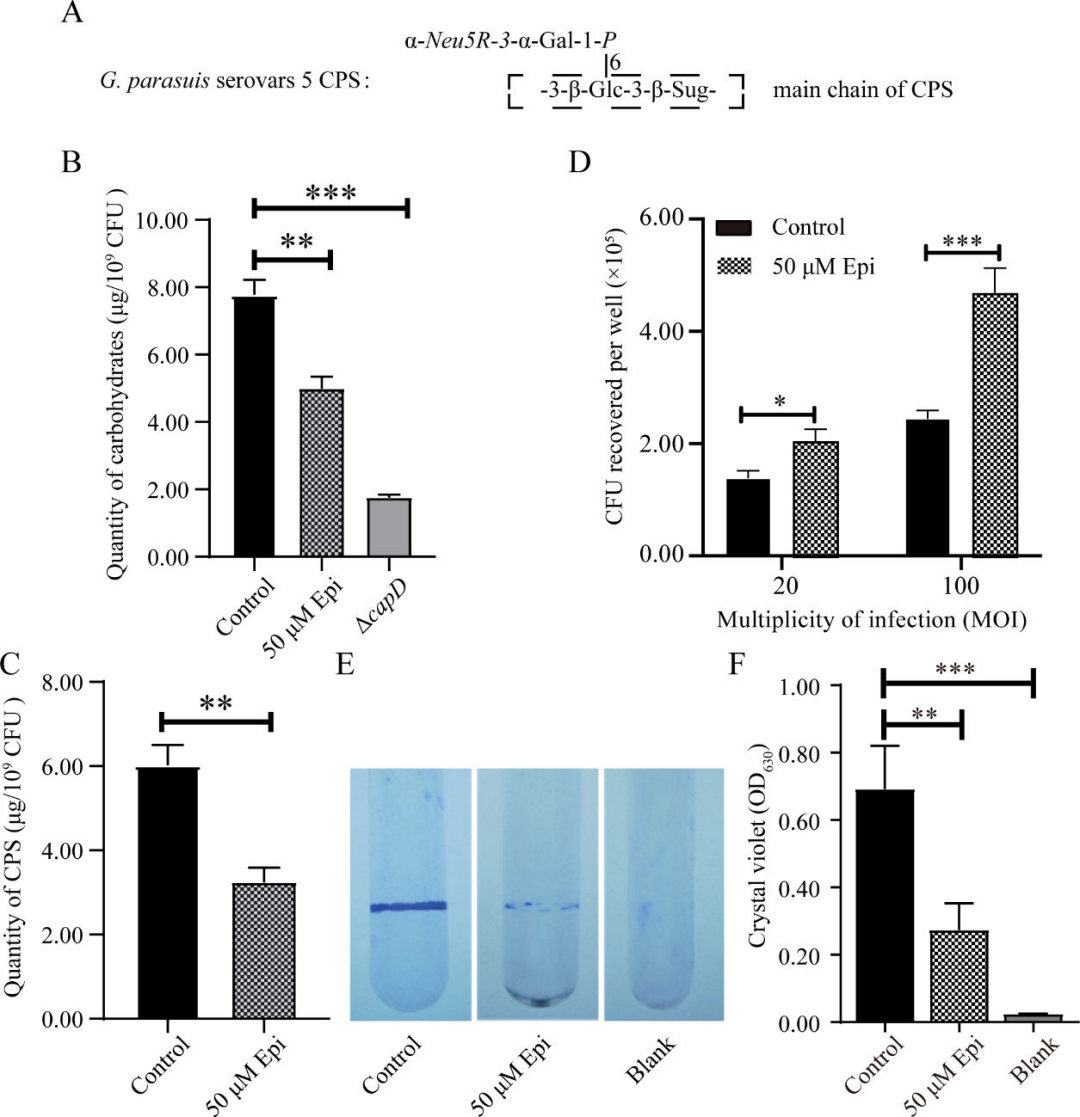
Examination of *G. parasuis* carbohydrate contents, adherence ability, and biofilm formation under Epi stimulation. (**A**) Structure of the capsular polysaccharide in *G. parasuis* serovar 5 (22). Sug, 2,4-diacetamido-2,4,6-trideoxy-ᴅ-galactopyranose; R, acetyl (Ac) or glycolyl (Gc); italic, non-stoichiometric components. (**B**) *G. parasuis* was cultured in TSB unsupplemented or supplemented with 50 µM Epi, and total carbohydrate content was determined and blanked with the *capD* deletion mutant Δ*capD* to accurately quantify the CPS (*n* = 3). (**C**) Epi stimulation dramatically inhibits CPS synthesis (*n* = 3). (**D**) The bacteria in logarithmic growth phase cultured in TSB unsupplemented or supplemented with 50 µM Epi were selected to interact with newborn pig tracheal epithelial cells at multiplicity of infection 20 and 100 for 2 h. The data represent the number of bacteria that adhered to the cells in each well of a 24-well plate (*n* = 3). (**E**) Biofilms of wild type cultured in the absence (control) or presence of 50 µM Epi at 48 h were stained with crystal violet. (**F**) Quantification of biofilm production (*n* = 3). Statistical analyses were performed using the one-way analysis of variance. In bar graphs, expression levels were expressed as mean ± standard error of the mean. The statistical significance of the indicated *P*-values was determined as *P < 0.05, **P < 0.01, and ***P < 0.001.

To determine whether Epi modulates the biosynthesis of CPS, we measured the total carbohydrate content in *G. parasuis* CF7066 cultured in tryptic soy broth (TSB) unsupplemented or supplemented with 50 µM Epi, respectively, and blanked with the CPS biosynthesis gene deletion mutant CF7066 Δ*capD* to accurately quantify the capsules. The relative content of total carbohydrate was assessed to quantitatively compare the difference in CPS formation. As shown in Fig. 1B and C, the addition of Epi resulted in significantly lower amounts of CPS. Subsequently, we observed changes in bacterial adhesion and biofilm formation in response to Epi stimulation, which are directly correlated with capsule polysaccharide content. The adherence ability of *G. parasuis* CF7066 significantly increased after Epi treatment (Fig. 1D), whereas the biofilm formation ability significantly decreased (Fig. 1E and F), both of which are consistent with a reduction of CPS synthesis. These results showed that the biosynthesis of CPS was considerably reduced under Epi stimulation, indicating that a novel regulatory mechanism underlies CPS synthesis in *G. parasuis*.

### The response regulator QseB self-regulates its own expression under Epi stimulation

**Fig 2:**
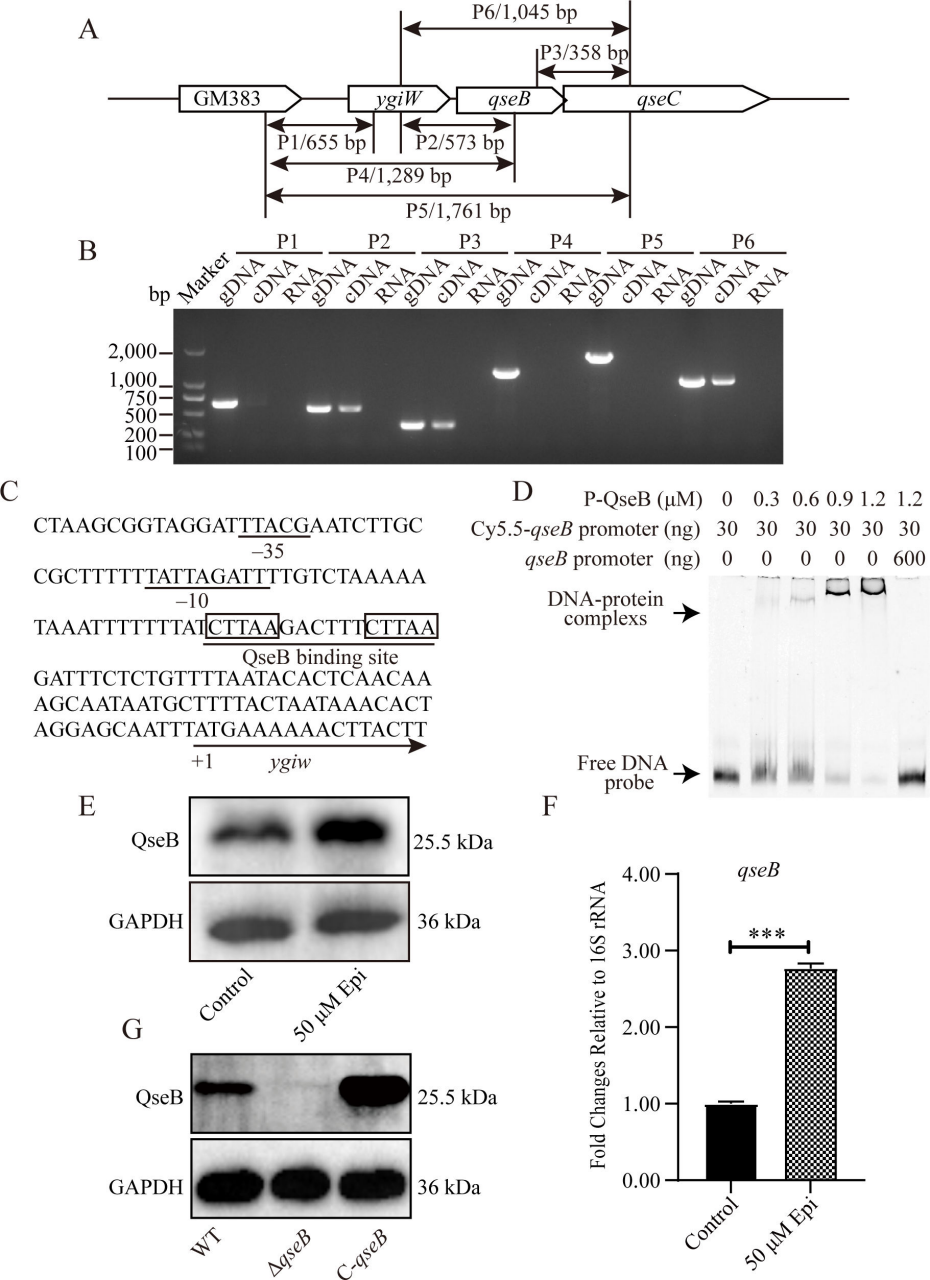
Exploration of the transcriptional mode of *qseB* and the positive regulation of *qseB* expression by QseB directly binding to target promoter in the presence of Epi. (**A**) Schematic diagram of primer design for *qseB* transcriptional analysis, the expected size of the PCR product is indicated at the bottom of the interval. (**B**) Reverse transcription polymerase chain reaction analysis of the transcription pattern of *qseB* in *G. parasuis*. (**C**) Nucleotide sequence of *qseB* operon promoter region. The underscore sequences are predicted –35 and –10, inside the rectangle is the predicted QseB binding site. (**D**) Labeled *qseB* promoter DNA sequences (30 ng) were incubated with different amounts of purified QseB protein (0 µM, 0.3 µM, 0.6 µM, 0.9 µM, and 1.2 µM) in the presence of acetyl phosphate. About 200-fold higher amount of unlabeled *qseB* promoter DNA sequences were used as specific competitors. The positions of protein-DNA complexes and free DNA probes were shown. (**E**) Western blot analysis to detect effect of Epi on QseB protein expression. (**F**) Quantitative real-time PCR analysis of the relative changes of *qseB* mRNA levels in the presence of Epi (*n* = 3). (**G**) Conﬁrmation of the deletion mutant Δ*qseB* and the complementary strain C-*qseB* using Western blot. The statistical significance of the indicated *P*-values was determined as *P < 0.05, **P < 0.01, and ***P < 0.001.

In order to explore the impact of Epi on the transcription and expression of *G. parasuis* genes, quantitative real-time PCR (qRT-PCR) and Western blot were used to determine mRNA and protein expression, respectively, and we found that the expression of *qseB* was significantly upregulated in the presence of 50 µM Epi (As shown in Fig. 2E and F).

To identify the full-length mRNA of the *qseB* operon, a reverse transcription polymerase chain reaction (RT-PCR) analysis was carried out. Using genomic DNA as a template, six gene-specific primers (Table 2) were used to create the 655 bp P1, 573 bp P2, 358 bp P3, 1,289 bp P4, 1,761 bp P5, and 1,045 bp P6 fragments (Fig. 2A). The corresponding primer sets were applied to run RT-PCR from the first strand of cDNA. As illustrated in Fig. 2B, the genes *ygiW*, *qseB*, and *qseC* were co-transcribed, and a schematic diagram of primer design is shown in Fig. 2A. Following that, we analyzed the *ygiW-qseBC* operon, the –35 element and the –10 element, and the putative QseB binding sequence (Fig. 2C). Electrophoretic mobility shift assay (EMSA) was performed, and the result showed that QseB could bind to its own promoter in a concentration-dependent manner, the addition of unlabeled probe can compete with the labeled probe, which further demonstrates the binding ability (Fig. 2D), whereas there was no interaction between QseB and the control probe (the promoter DNA of kanamycin-resistance gene).

To investigate the detailed biological function of the adrenaline response regulatory system QseBC in *G. parasuis,* the deletion mutant Δ*qseB* was obtained by homologous replacement, and the complementary strain C-*qseB* was constructed by electro-transforming the plasmid pSHGB into the Δ*qseB*. Western blot analysis revealed that the QseB levels in the C-*qseB* were signiﬁcantly higher than that in the wild type (WT), whereas the Δ*qseB* did not express QseB. These findings indicated that the mutant strains had been successfully constructed (Fig. 2G).

### QseB can directly interact with the *capD* gene promoter

**Fig 3:**
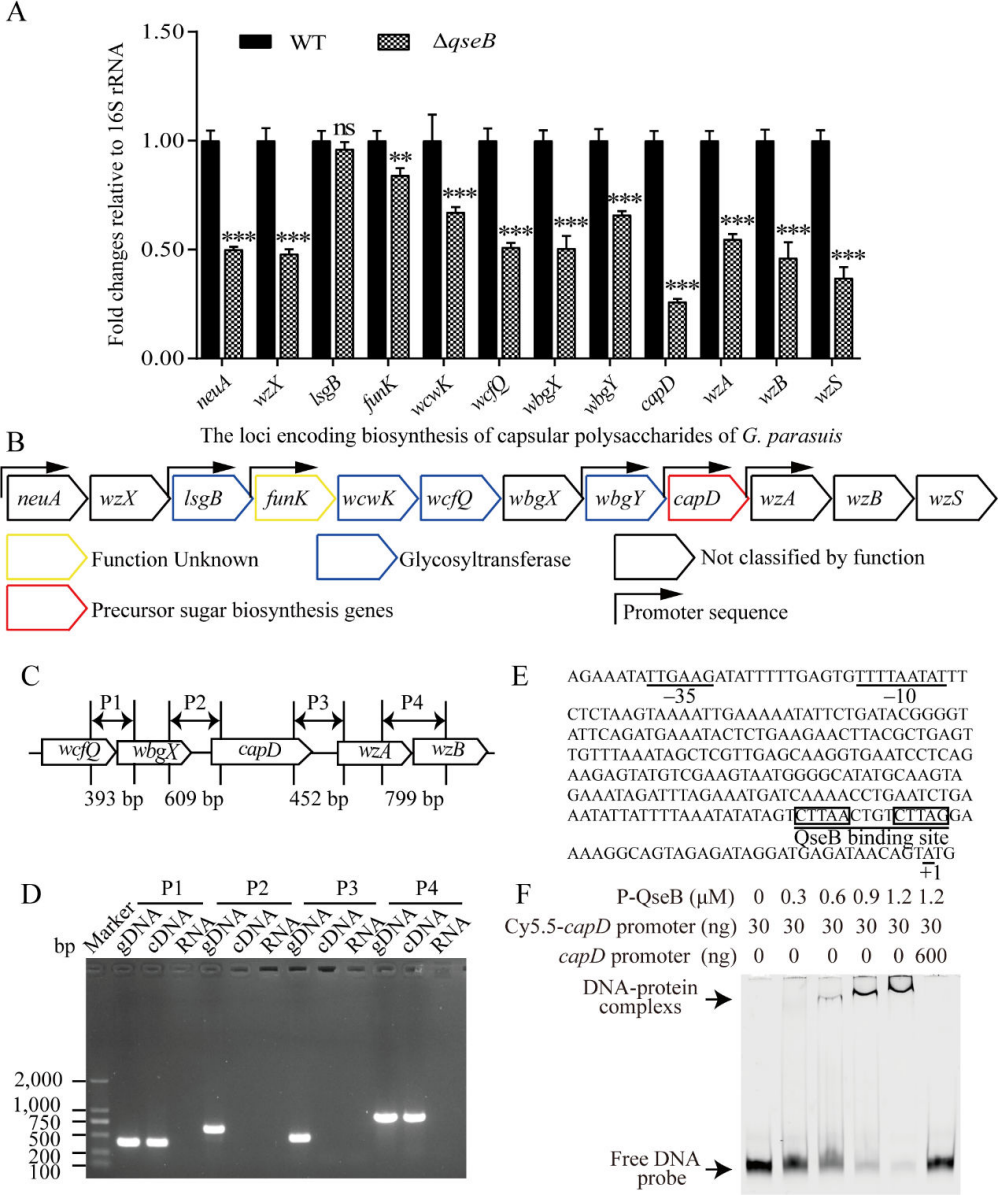
*G. parasuis* QseB could directly bind to the target promoter region of *capD*. (**A**) Quantitative RT-PCR was used to measure the changes of genes related to capsular polysaccharide in *G. parasuis* Δ*qseB* strain (*n* = 3). (**B**) Schematic diagram of capsule locus in *G. parasuis* CF7066. Direction of CDS is represented by the direction of the arrow; the gene name assigned is inside the arrow. Coloring represents the predicted function of the gene. Arrows above the CDSs represent predicted promoters from BPROM output. (**C**) Schematic diagram of primer design for *capD* transcriptional analysis, the expected size of the PCR product is indicated at the bottom of the interval. (**D**) RT-PCR was carried out to analyze the transcription pattern of capsular polysaccharide gene cluster of *G. parasuis*. (**E**) Nucleotide sequence of *capD* operon promoter region. The underscore sequences are predicted –35 and –10, inside the rectangle is the predicted QseB binding site. (**F**) Labeled *capD* promoter DNA sequences (30 ng) were incubated with different amounts of purified QseB protein (0 µM, 0.3 µM, 0.6 µM, 0.9 µM, and 1.2 µM) in the presence of acetyl phosphate. About 200-fold higher amounts of unlabeled *capD* promoter DNA sequences were used as specific competitors. The positions of protein-DNA complexes and free DNA probes were shown. Statistical analyses were performed using the two-way analysis of variance. In bar graphs, expression levels were expressed as mean ± standard error of the mean. The statistical significance of the indicated *P*-values was determined as *P < 0.05, **P < 0.01, and ***P < 0.001.

The results presented above suggested that QseB may participate in the Epi-mediated regulation of polysaccharide synthesis. The transcription of CPS genes was then examined by qRT-PCR to determine the possible effects caused by the deletion of *qseB*. All 12 genes in the CPS gene cluster except *lsgB* were down-regulated in Δ*qseB* strain compared to in WT, among which the *capD* was the most significantly down-regulated gene (Fig. 3A).

We predicted the promoter of *G. parasuis* CF7066 in BPROM (http://www.softberry.com/berry.phtml?topic=bprom), and found that both the *lsgB,* which had no transcriptional change in Δ*qseB* strain, and the *capD*, which had the largest change, were independently transcribed (Fig. 3B). At the same time, we predicted one putative promoter at 194 bp from the translation start codon of *capD* gene (Fig. 3E). The transcription of *capD* gene was then analyzed using RT-PCR. Figure 3C and D show that the primer combination P2 was unable to amplify the 609 bp DNA fragment covering partial *wbgX* and *capD* genes, meanwhile, the primer combination P3 failed to produce the 452 bp DNA fragment containing partial *capD* and partial *wzA* genes. These results demonstrated that the *capD* was transcribed as a single transcription unit.

In addition, the upstream region of *capD* in *G. parasuis* was searched for the *E. coli* consensus sequence for the QseB binding site. In the *capD* promoter region, one QseB binding sequence (CTTAAN4CTTAG) was discovered in *G. parasuis* (Fig. 3E). To confirm whether this is a genuine QseB binding site, the promoter fragment was incubated with *G. parasuis* QseB and then analyzed *via* EMSA. As shown in Fig. 3F, the *capD* promoter fragment bound to QseB while competition by an unlabeled DNA probe attenuated the fluorescent signal of the QseB-DNA complex, suggesting that the interaction of QseB with the *capD* promoter region was specific. These findings proved that QseB can directly bind to the promoter region of *capD*.

### The *capD* is related to adherence and biofilm formation

**Fig 4:**
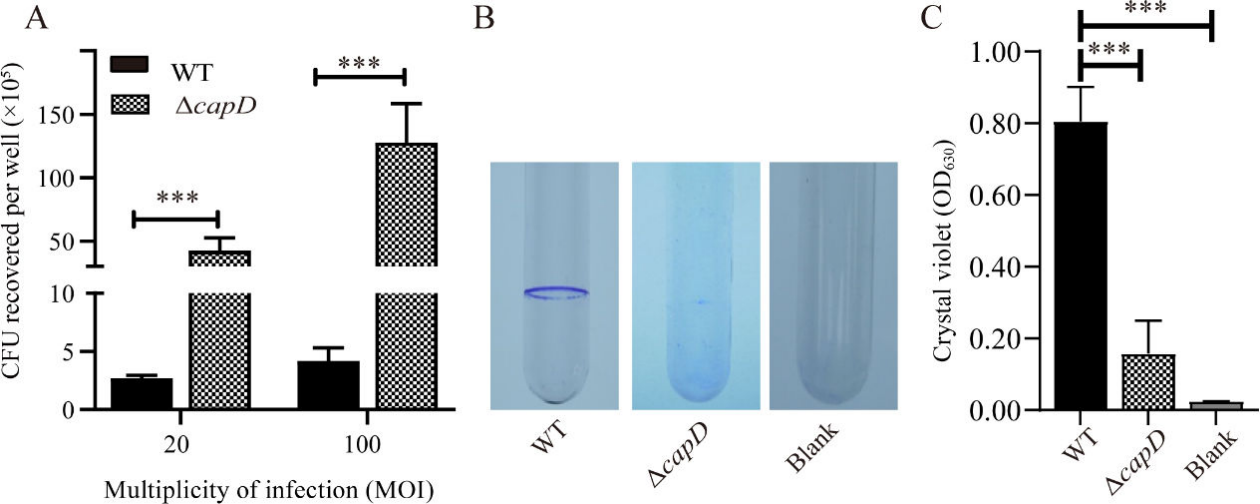
*G. parasuis capD* was related to capsular polysaccharide synthesis, adhesion, and biofilm formation ability. (**A**) NPTr cells were inoculated with the indicated strains at multiplicity of infection 20 or 100, culture plates were incubated for 2 h. The data represent the number of bacteria that adhered to the cells in each well of a 24-well plate (*n* = 3). (**B**) Biofilms of WT and Δ*capD* at 48 h were stained with crystal violet. (**C**) Quantification of biofilm production. Statistical analyses were performed using the one-way analysis of variance. In bar graphs, expression levels were expressed as mean ± standard error of the mean (*n* = 3). The statistical significance of the indicated *P*-values was determined as *P < 0.05, **P < 0.01, and ***P < 0.001.

A polysaccharide biosynthesis protein that was connected to *G. parasuis* pathogenicity is encoded by the *capD* gene. In the present study, a *capD* gene deletion strain was constructed and the relationship between *capD* and the adherence and biofilm formation ability was analyzed. Additionally, the absence of *capD* made *G. parasuis* more likely to adhere to newborn pig tracheal epithelial (NPTr) cells (Fig. 4A), and the Δ*capD* essentially lost the ability of biofilm formation (Fig. 4B and C). It demonstrated that the lack of *capD* gene had a negative impact on biofilm formation, as well as increased adherence.

### QseB affects the formation of *G. parasuis* CPS *via* interacting with *capD*

**Fig 5:**
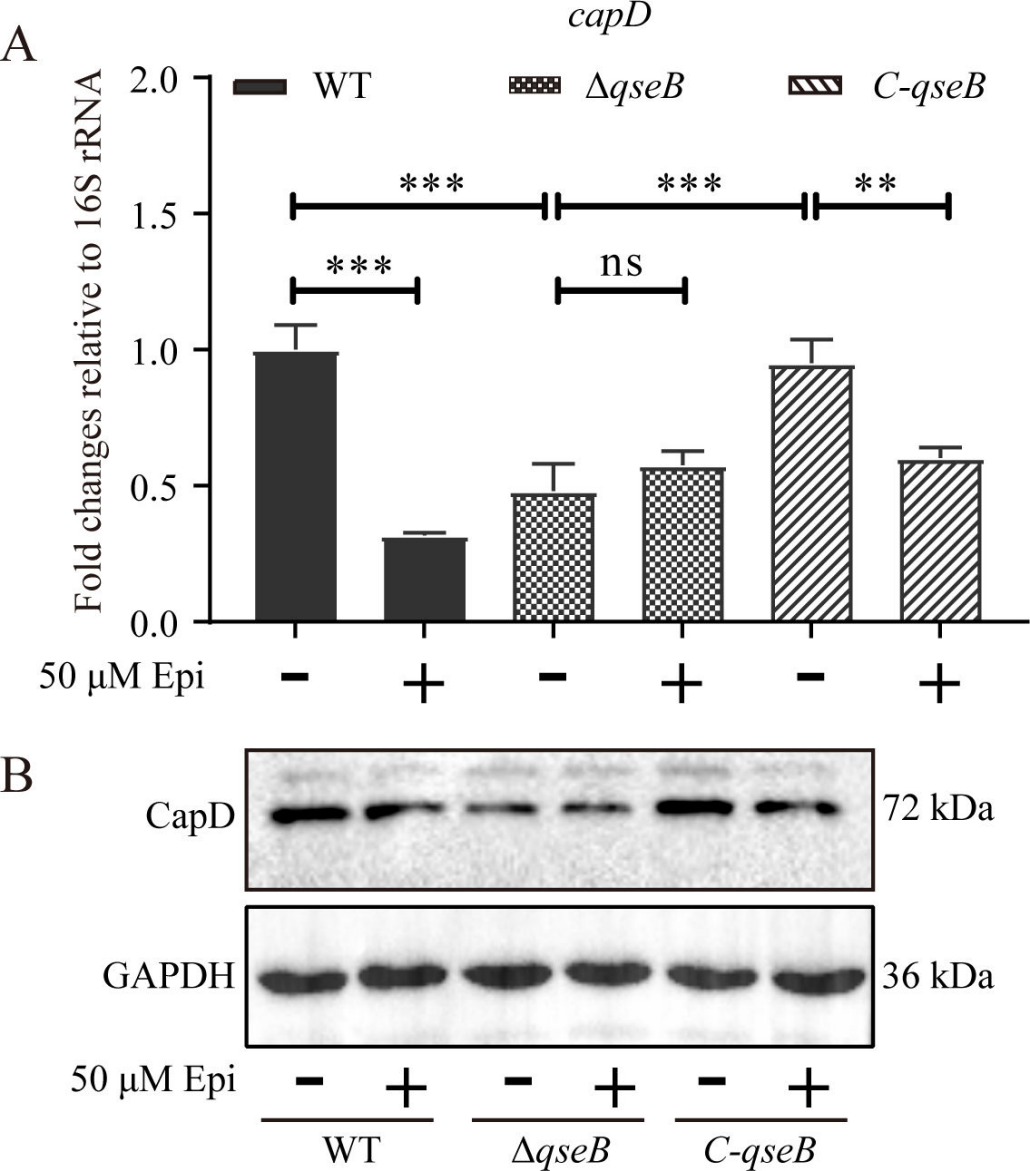
*G. parasuis* QseB negatively regulates *capD* in the presence of 50 µM Epi. (**A**) The expression levels of *capD* in strains WT, Δ*qseB*, and C-*qseB* with 50 µM Epi stimulation were analyzed by qRT-PCR (*n* = 3). (**B**) CapD levels in strains WT, Δ*qseB*, and C-*qseB* were analyzed by Western blot using polyclonal antibodies raised against CapD. Protein extracts (100 µg) were obtained from bacteria in logarithmic growth phase cultivated in TSB unsupplemented or supplemented with 50 µM Epi. In bar graphs, expression levels were expressed as mean ± standard error of the mean. The statistical significance of the indicated *P*-values was determined as *P < 0.05, **P < 0.01, ***P < 0.001, and *P* > 0.05 ns (not significant).

Based on the combination of QseB directly binding to the *capD* promoter with a putative QseB binding motif located in *capD* promoter region, we speculated that *G. parasuis* regulated the expression of *capD* through QseB in the presence of Epi. In order to test this hypothesis, qRT-PCR was performed to analyze relative expression levels of *capD*. The mRNA levels in various strains and conditions were normalized with the concentration of the 16S rRNA gene. As shown in Fig. 5A, whether there was Epi, the mRNA levels of *capD* in Δ*qseB* were significantly lower than that in the WT and the complementary strain (C-*qseB*). Additionally, following Epi stimulation, the *capD* transcription was significantly inhibited in WT and C-*qseB*.

Then, we examined changes in CapD expression at the protein levels. In order to prepare immune serum, we first expressed recombined protein rCapD without signal peptide (1–168 aa) in *E. coli* BL21 and immunized mice. Subsequently, Western blot was used to determine the expression levels of CapD in different strains following Epi treatment. Unsurprisingly, the changing trend of protein levels was consistent with that of the mRNA transcriptional levels (Fig. 5B). The CapD levels in the Δ*qseB* strain were considerably decreased and were recovered to WT levels in the C-*qseB* strain. Aside from that, the transcription of *capD* was significantly down-regulated in both the WT strain and C-*qseB* strain under Epi stimulation. These results reveal that *G. parasuis* negatively regulates the expression of CapD in the presence of 50 µM Epi, and the *qseB* gene is crucial for both Epi stimulation and *capD* transcription.

## DISSCUSSION

*G. parasuis* is a commensal bacterium in the upper respiratory tract of healthy pigs, and it can invade piglets and cause Glässer’s disease under stress conditions (23, 24). However, the pathogenic mechanism is still unclear. In the present study, the regulation of capsule polysaccharide expression in *G. parasuis* was explored for its biosynthesis changes in the presence of Epi. The results revealed that CPS expression was inhibited in the presence of Epi and we further found that the QseBC two-component system negatively regulated the synthesis of CPS in response to Epi. Overall, this study initially explored the pathogenesis of *G. parasuis* following the perception of Epi.

In some cases, stress can have adverse effects on a variety of immunological mechanisms, alter bacterial pathogenicity, and ultimately affect disease progression (25, 26). Epi is the major signaling molecule during stress and its secretion shifts bacteria from a commensal to an invasive pathogenic state (27). Previous studies have shown that stress can result in elevated Epi levels, which can influence bacterial pathogenicity in a variety of ways, such as boosting bacterial growth, improving bacterial adhesion to host tissue, recognizing the host environment, and promoting the expression of virulence factors (28 – 30). As the primary quorum-sensing system of bacteria, QseBC can sense and respond to Epi and regulate bacterial growth, motility, biofilm formation, and virulence gene expression (31). In the current investigation, Epi stimulation significantly increased *G. parasuis* CF7066’s ability to adhere to host cells and significantly decreased its capacity to develop biofilms.

The initial phase of the pathogenesis of mucosal microorganisms is commonly associated with colonization, followed by intimate contact with host cells. The reduced amount of capsule polysaccharide leads to the exposure of adhesive molecules, which promotes colonization and uptake. Previous studies have demonstrated that bacterial adherence to host epithelial cells plays an essential role in causing infections (32). The displacement of *Kingella kingae* capsule could expose a trimeric autotransporter adhesin called Knh and allows Knh to mediate high-strength adherence to the host cell (33). In previous studies, CPS has been shown to reduce adherence of enterotoxigenic *Escherichia coli* to isolated intestinal epithelial cells of pigs (34). In the present study, Epi stimulation dramatically inhibited CPS expression, and both Epi stimulation and *capD* deletion enhanced the adherence of *G. parasuis* to host cells, which was as hypothesized.

The quorum-sensing system seemed to be closely related to the bacterial CPS synthesis; much evidence revealed that quorum-sensing system signals such as Fe^3+^ and AI-2 regulates CPS synthesis (35). In the present study, *G. parsuis* CF7066 senses Epi *via* QseBC TCS, which is consistent with a previous study in which the QseC of *G. parsuis* MY1902 might sense Epi in the environment and thus regulate bacterial density (36). Additionally, the QseC sensor kinase was a bacterial adrenergic receptor (15). In the present study, quantitative real-time PCR analysis was performed to identify the potential target genes of QseBC regulatory network in *G. parasuis* CPS biosynthesis loci. Interestingly, in Δ*qseB*, the genes involved in capsular polysaccharide were significantly down-regulated in comparison to the control, with the exception of the *lsgB* gene, which encodes a sialyltransferase. It is worth noting that the deletion of *qseB* resulted in the most pronounced reduction in *capD* expression. CapD is an integral membrane protein, which catalyzes the first steps in the synthesis of the soluble capsule precursors UDP-D-FucNAc, and the *capD* mutant had a significantly higher cell surface hydrophobicity than the wild-type and the complementary strain, resulting in less biofilm production (37) and reduced CPS production (38). In a word, the present study showed that the expression of CPS synthesis gene *capD* in *G. parsuis* was regulated *via* QseBC two-component system. Under the presence of Epi, QseB can bind to the promoter of *capD* thereby inhibiting its expression. As a result, the synthesis of CPS was significantly decreased, and the biofilm formation was significantly repressed, making it easier for the bacteria to adhere to host epithelial cells. This may be the main mechanism by which *G. parasuis* transitions from symbiosis to infection and invades piglets under the action of Epi.

**Fig 6:**
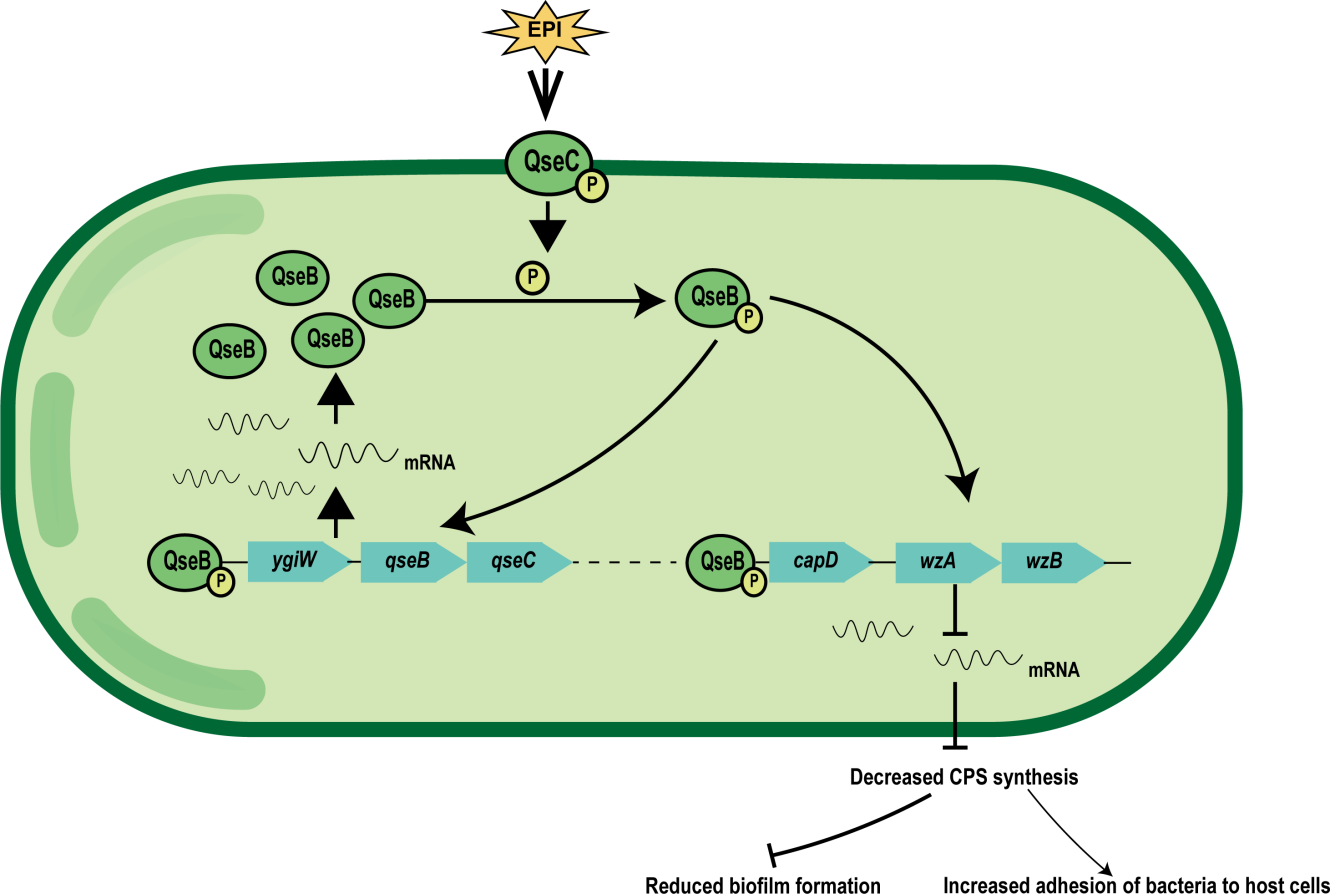
Model demonstrating that QseB negatively controls CPS synthesis in response to Epi stimulation. Under Epi stimulation, phosphorylated QseB regulates CPS synthesis by binding to the *capD* promoter and repressing its transcription.

In conclusion, we found that in the presence of Epi, the heavily phosphorylated QseB can interact with the capsular polysaccharide synthesis-related gene *capD*, leading to changes in bacterial adhesion and biofilm formation (Fig. 6). In the present study, we initially investigated the regulatory function of the QseBC two-component system in capsule polysaccharide synthesis, biofilm formation, and adhesion capability under Epi stimulation. Therefore, QseBC two-component system may be a vaccine or drug target candidate for the development of novel antibacterial to treat *G. parasuis* infections.

## MATERIALS AND METHODS

### Bacterial strains, plasmids, and growth conditions

The bacterial strains used in this study and their sources are listed in Table 1. *G. parasuis* and its isogenic derivatives were cultured on tryptic soy agar (TSA) or in TSB (Difco Laboratories, Detroit, MI, USA) supplemented with 5% inactivated bovine serum (Tianhang, Hangzhou, Zhejiang, China) and 10 µg/mL nicotinamide adenine dinucleotide (BioFroxx, Einhausen, Germany). *E. coli* DH5α and BL21 (DE3) were grown in Luria-Bertani (LB) medium (Difco Laboratories, Detroit, MI, USA) at 37°C. Agar was added to the medium at a concentration of 1.5% when a solid medium was desired. When necessary, 50 µg/mL of kanamycin or 20 µg/mL of gentamicin was added to the medium.

**TABLE 1:** Bacterial strains and plasmids used in this study^*a*^

Strain or plasmid	Description or function	Source or reference
*G. parasuis* CF7066 (WT)	*G. parasuis* serovar 5 strain	This laboratory
CF7066Δ*qseB::kan* (Δ*qseB*)	CF7066 with deletion of *qseB*, Kan^R^	This study
CF7066Δ*capD::kan* (Δ*capD*)	CF7066 with deletion of *capD*, Kan^R^	This study
CF7066Δ*qseB/qseB::kan/gen* (C*-qseB*)	Δ*qseB* complemented with *qseB*, Kan^R^, Gen^R^	This study
*E. coli* DH5α	Cloning host	Novagen
*E. coli* BL21 (DE3)	Expression host	Novagen
pK18mobsacB	Mobilizable suicide vector, Kan^R^	(39)
pSHG3	*E. coli-G. parasuis* shuttle vector, Gen^R^	(40)
pKBukd	A 1,903 bp overlap fragment containing Kan^R^, and the upstream and downstream fragment sequences of the *qseB* gene in pK18mobsacB, Kan^R^	This study
pKDukd	A 1,961 bp overlap fragment containing Kan^R^, and the upstream and downstream fragment sequences of the *capD* gene in pK18mobsacB, Kan^R^	This study
pSHG3-*qseB*	pSHG3 vector containing deleted *qseB* gene and corresponding promoter for complementation	This study
pET-28a	Expression vector, Kan^R^	Novagen
pET-QseB	pET-28a vector containing QseB encoding sequence	This study
pET-CapD	pET-28a vector containing CapD encoding sequence	This study

^*a*^ WT, wild-type strain; Kan^R^, kanamycin resistance; Gen^R^, gentamicin resistance.

### Construction of gene deletion and complementary strains

The mutant strains Δ*qseB* and Δ*capD* used in this study were derivatives of *G. parasuis* serovar 5 strain CF7066. These two mutant strains were constructed by natural transformation as previously described (41). In brief, the flanking fragments of the target gene and the kanamycin resistance cassette from plasmid pSHK3 were cloned into the pK18mobsacB vector to generate the recombinant plasmids pKBukd and pKDukd, respectively. Each recombinant plasmid (pKBukd or pKDukd) was then introduced into *G. parasuis* CF7066 *via* bacterial natural transformation.

All complementary strains were constructed by electro-transforming the shuttle vector pSHG3 containing the target gene. To obtain the complementation plasmid carrying a target gene, the promoter sequence was analyzed using prediction bacterial promoter (http://linux1.softberry.com). A DNA fragment containing *qseB* and its promoter was amplified from CF7066 genomic DNA using PCR. The PCR product was then cloned into pSHG3 plasmid to generate pSHG3-*qseB*. Subsequently, the recombinant plasmid was introduced into Δ*qseB* by electroporation. The complementary strains were screened on TSA plates supplemented with 20 µg/mL gentamicin and confirmed by PCR and sequencing.

### Quantitative analysis of polysaccharides

Previous research has revealed that CPS of *G. parasuis* had the main chain -3-β-Glc6*P*-3-β-Sug- (Fig. 1A) (22). The anthrone assay was used to analyze the synthesis of the CPS as described previously (42) with some modifications. The strains in the logarithmic growth period [optical density at 600 nm (OD_600_) ≈ 0.6] were harvested by centrifugation at 8,000 × *g* for 10 min at 4°C, washed with 0.01 M Phosphate buffered saline (PBS) three times and 150 mM Tris-HCl (pH 7.0) once. The bacteria were then resuspended with 2 mL 150 mM Tris-HCl (pH 7.0). Following that, the bacterial solution was mixed with anthrone-sulfuric acid reagent at a volume ratio of 1:3 and heated at 100°C for 15 min. After placing all of the samples on ice, absorbance measurements were taken at 620 nm using a FLUOstar Omega microplate reader, and the amounts of CPS were determined from a standard curve of glucose. CPS concentrations are expressed as micrograms per 10^9^ colony forming units (CFU), and the experiments were performed in triplicate.

### Adhesion assay

The adhesion assay was used to count the total number of cell-associated (intracellular plus surface-adhered) bacteria, as previously described (43). Bacteria were pelleted, washed twice with PBS, and resuspended in fresh cell culture medium without antibiotics. NPTr cells were cultured as a monolayer in Dulbecco’s Modified Eagle Medium (DMEM) supplemented with 10% (vol/vol) heat-inactivated fetal bovine serum. Cells were infected with *G. parasuis* suspensions at a multiplicity of infection of 20 and 100, respectively, after being cultured to 80%–90% confluency in 24-well plates. The plates were centrifuged at 800 × *g* for 10 min to promote bacterial contact with the cell monolayer surface and incubated for 2 h at 37°C in 5% CO_2_ to allow bacterial adherence. Subsequently, non-attached cells were removed by washing the cell monolayers with PBS five times. After washing, 0.1% Triton X-100 was added to each well to lyse the NPTr cells. The lysates were serially diluted in PBS and plated on TSA plates to count the CFU after 48-h incubation at 37°C.

### *In vitro* biofilm formation assay

A method that has already been described (44) was modified in order to examine the biofilm-formation ability. Briefly, when the cultures had grown to the mid-exponential phase, 50 µL of the bacterial solution was transferred to borosilicate glass tubes containing 2 mL of TSB medium, and then incubated vertically with circular agitation (150 rpm) at 37°C for 48 h. To visualize biofilms, the contents of the tubes were sucked with an injector for staining, and the tubes were washed three times with 2 mL of sterile PBS to remove loosely adhering cells. After being air‐dried, the tubes were dyed with 2 mL of 1% crystal violet solution for 10 min at room temperature. With the use of an injector, the dye solution was extracted from the tubes. The tubes were then thoroughly washed under running tap water, and extra water was removed from the wells as described above. The adherent cells were lysed with 200 µL of 33% (vol/vol) glacial acetic acid, and 100 µL of the solution was transferred to a microtiter plate to obtain the OD of each tube at 630 nm. The findings of each test were averaged after being run three times.

### Reverse transcription PCR and quantitative real-time PCR

To obtain reliable results, methods of bacterial total RNA purification (45) and qRT-PCR (46) were applied. In brief, the overnight grown bacterial suspensions were inoculated into fresh medium at a ratio of 1:50 with circular agitation (200 rpm) for 6 h at 37°C until logarithmic growth period (OD_600_ ≈ 0.6). Collect the bacteria after mixing twice the volume of RNAprotect Bacteria Reagent (Qiagen, Hilden, Germany) with the bacterial suspension for 10 min at room temperature. Later, total bacterial RNA was extracted using TRIzol (Invitrogen, Carlsbad, CA, USA) according to the manufacturer’s instructions. The total RNA concentration was quantified by measuring the ratio of OD_260_/OD_280_ and then analyzed by gel electrophoresis to determine whether it was intact. After removing the residual genomic DNA with a gDNA wiper (Vazyme Biotech, Nanjing, Jiangsu, China), the cDNA was synthesized with random hexamer primers by using a HiScript III 1st Strand cDNA Synthesis Kit R312 (Vazyme Biotech, Nanjing, Jiangsu, China) according to the manufacturer’s instruction.

Quantitative real-time PCR was performed using AceQ qPCR SYBR Green Master Mix (Vazyme Biotech, Nanjing, Jiangsu, China) on a Bio-Rad CFX96 machine (Bio-Rad Company, Pleasanton, CA, USA). The 16S rRNA gene of *G. parasuis* from the same sample was used as an endogenous reference and the relative copy number of the target gene’s mRNA was calculated using the comparative cycle threshold (2^–ΔΔCT^) method. All data came from at least three independent biological experiments. The primer sequences used for gene expression analysis are listed in Table 2.

**TABLE 2:** Primers used in this study^*a*^

Oligonucleotide	Sequence	Description
Δ*qseB*-upF	CGCggatccACCGCTTGTATTTCTCTGTTTTAATACACTCAAC	Used for construction of marker-exchange mutant of *qseB* and *capD* by pK18mobsacB, respectively.
Δ*qseB*-upR	TATTTTTATCTTGTGCAATGCTCAACCTCCAAATTCTAAAAGA	
Δ*qseB*-downF	TAATCAGAATTGGTTAATTGATGAAGTTGCTTAAAAATACCAGTT	
Δ*qseB*-downR	GCtctagaACAAGCGGTGGCTAAACACCATTCCTTGAA	
Δ*capD*-upF	CGgaattcACCGCTTGTTATTGATAACGCTTAATTTGTTGT	
Δ*capD*-upR	TATTTTTATCTTGTGCAATGTATATTTAGATTAGCCTATTCCTTT	
Δ*capD*-downF	TAATCAGAATTGGTTAATTGACTGTTATCTCATCCTATCTCTACT	
Δ*capD*-downR	CGCggatccACAAGCGGTGAAAGTGAAATTTTGGGTAAAT	
*kan*-F	CATTGCACAAGATAAAAATA	
*kan*-R	CAATTAACCAATTCTGATTA	
C-*qseB*-F	CCGctcgagAGCGGTGAGATCACGGTGG	Used for construction genetic complementary vector of *qseB*.
C-*qseB*-R	CGggatccTTAAGCAACTTCATCGTTTTTTCC	
*qseB*-1F	GGTCGCCAATGGAAAACAAC	Used for RT-PCR to analyze *ygiW-qseB-qseC* operon. *qseB*-1F/1R, *qseB*-2F/2R, *qseB*-3F/3R, *qseB*-1F/2R, *qseB*-1F/3R, and *qseB*-2F/3R primers for 655 bp P1, 573 bp P2, 358 bp P3, 1,289 bp P4, 1,761 bp P5, and 1,045 bp P6, respectively.
*qseB*-1R	CCAACTTGCCTTGTAATACGACA	
*qseB*-2F	TATTGACGATGATGCTTGGAGAG	
*qseB*-2R	TGTTCAATCACAGGGTTGGTATG	
*qseB*-3F	TGTTCTCACCCGTTCTTTCATTG	
*qseB*-3R	CGTTCCATCAATAATGTGCGTAA	
*capD-*1F	AGAAGATTGTGGCATTACTCGT	Used for RT-PCR to analyze *capD* operon. *capD*-1F/1R, *capD*-2F/2R, *capD*-3F/3R, and *capD*-4F/4R for 393 bp P1, 609 bp P2, 452 bp P3, and 799 bp P4, respectively.
*capD-*1R	CTACACGAACCTGCCTAAA	
*capD-*2F	TGGAGTGATTTTGTTCTCATTC	
*capD-*2R	CCGCAATAGCAGAAACTAAAAG	
*capD-*3F	AATTGGCTTGTCAGCATAATGAATG	
*capD-*3R	TCATCCCACACCCAGAAAG	
*capD-*4F	GAATCGTTTGTTATCTGATGGC	
*capD-*4R	GCTATCCAACCAATGACCAA	
E*-qseB-*F	Cy5.5-TTATTGGGGCAAACTATAGATTC 194	Used for amplify DNA probe of *qseB* (194 bp) and *capD* (194 bp) in EMSA assay.
E*-qseB-*R	Cy5.5-AAATTGCTCCTAGTGTTTATTAGTA	
E*-capD-*F	Cy5.5-GAAGAACTTACGCTGAGTTG 194	
E*-capD-*R	Cy5.5-ACTGTTATCTCATCCTATCTCTACT	
P*-qseB-*F	CGggatccATGCGTATTTTATTAATTGAAGACG	Used for construction of pET28a::*qseB* vector.
P*-qseB-*R	CCGctcgagTTTAAGCAACTTCATCGTTTTTTC	
P*-capD-*F	CGCggatccCGTTCCCATGATTATTTGC	Used for construction of pET30a::c*apD* vector, truncated protein (168–646 aa).
P*-capD-*R	CCCaagcttGTTTATCTACTTGCATCCGCC	
m-16S rRNA-F	ACACTGGAACTGAGACAC	Used for relative qRT-PCR to check the mRNA transcription levels of corresponding genes.
m-16S rRNA-R	GTGCTTCTTCTGTGACTAAC	
m-*neuA*-F	CCTGAACGTAATGGACTA	
m-*neuA*-R	CCTGTAGAATTAGCTCAAAG	
m-*wzX*-F	CGCATACAAACCAAGATC	
m-*wzX*-R	GCGAAAGACGGTTACTAA	
m-*lsgB*-F	GCTCCACTATAGAACGTATA	
m-*lsgB*-R	GAGAGAGGTTTATTATTGATGG	
m-*funK*-F	GTCGATTTACCATCTTGAA	
m-*funK*-R	TTCCCCTCTTATTCTTGATA	
m-*wcwK*-F	CTGAGAACAAAGTGTTCTTA	
m-*wcwK*-R	TGAGGTTAGTTAATAGTCAATG	
m-*wcfQ*-F	ACAGCATACACATTTTCTC	
m-*wcfQ*-R	CCCTGTTATGGTTTGAAG	
m-*wbgX*-F	AGAGCGAGATGTAATCCA	
m-*wbgX*-R	CGAAGAAGAAATTAATGAAGTTG	
m-*wbgY*-F	TACCCAAAATTTCACTTTCATC	
m-*wbgY*-R	AGACGAGTTACCTCAGTTA	
m-*capD*-F	AAGGGAGTGTTTCTATCAATGATG	
m-*capD*-R	GCGAATAATCTGCCGACAA	
m-*wzA*-F	CGAGTAATAGTCACATTATGC	
m-*wzA*-R	TTGCCTCATACCAATCTAA	
m-*wzB*-F	CTGCATCCTCAATCAATG	
m-*wzB*-R	GTCTGAACTATGTAAGAATGTAG	
m-*wzS*-F	TGAGCTTTATTCTCTCCATTA	
m-*wzS*-R	TTGCGATTTCTGAATTAGAG	

^*a*^ The restriction sites in the primers are marked in lowercase; letters are underlined to indicate the uptake signal sequences (USS) of *G. parasuis*.

RT-PCR was performed to detect the full-length mRNA of *qseB* and *capD* operons. Specific primers (Table 2) were designed based on genome sequence information, and genomic DNA and RNA were used as positive and negative controls, respectively.

### EMSA

EMSA was carried out in the same way as described in a previous study (47) with some modifications. Briefly, the 194 bp upstream promoter region of the *capD* gene and *qseB* gene was amplified, respectively, by PCR with labeled primers to generate the Cy5.5-*capD* and Cy5.5-*qseB* promoter fragment from CF7066-WT genomic DNA. The amplified fragment was purified using the Gel Extraction Kit (Omega Bio-Tek Inc., Norcross, GA, USA). The purified recombinant protein QseB (rQseB) was dialyzed against binding buffer (50 mM Tris, 10 mM MgCl_2_, 0.1 mM dithiothreitol (DTT), pH 7.0). Then, 30 ng of the labeled probe and increasing amounts of rQseB were incubated for 30 min at room temperature in binding buffer; 20 mM lithium potassium acetyl phosphate (Sigma-Aldrich, Steinheim, Germany) was added to the reaction system. Unlabeled PCR products (600 ng) were used as competitive probes in competitive experiments. After adding 10% (vol/vol) volume of loading buffer (0.25% bromophenol blue, 0.25% xylene cyanol, 30% glycerol) to the reaction mixtures, electrophoresis was performed on a 6% polyacrylamide gel in 0.5× Tris-Borate-Ethylenediaminetetraacetic acid (TBE) buffer (45 mM Tris, 45 mM boric acid, 0.5 mM EDTA, pH 8.0) at 4°C and 100 V for 1 h, and the gels were exposed to an Amersham Typhoon 5 (GE HealthCare, CA, USA).

### Recombinant protein expression and purification

The *qseB* and 1,437 bp sequence omitting the signal peptide sequence of *capD* were amplified by PCR using the rQseBF/R and rCapDF/R primers (Table 2) and CF7066 genomic DNA as the template, respectively. The PCR products were gel purified using the Gel Extraction Kit (Omega Bio-Tek Inc., Norcross, GA, USA). Then, the amplified fragments were digested with restriction enzymes *Bam*HI/*Xho*I (New England Biolabs, Ipswich, MA, USA) or *Bam*HI/*Hin*dIII (New England Biolabs, Ipswich, MA, USA), and ligated to the similarly digested pET28a. The recombinant plasmids were designated as pET-*qseB* and pET-*capD* and were introduced into *E. coli* BL21 (DE3) (Transgen Biotech, Beijing, China).

*E. coli* BL21 (DE3) cells containing recombinant plasmid were respectively cultured in LB medium in a shaking incubator at 37°C. When the cultures were grown to the OD_600_ values of 0.4–0.5, isopropyl-β-d-thiogalactoside was then added at a final concentration of 0.8 mM to induce expression at 37°C for 5 h. The bacteria were harvested by centrifugation at 12,000  × *g* for 5  min and washed with PBS three times. The harvested cells were suspended in binding buffer and broken by passing through a pressure cell disruptor three times. The rQseB was purified as previously described (48) by Nickel nitrilotriacetate (Ni-NTA) resin affinity chromatography (Qiagen, Hilden, Germany). However, the CapD protein was expressed as inclusion body, from which correctly folded protein can be easily extracted using nondenaturing solvents (49). The purified protein was confirmed by Western blot with an anti-His tag antibody (ABclonal, Wuhan, Hubei, China).

### Western blot analysis

Antisera against QseB and CapD proteins were raised in mice using recombinant proteins. The BALB/c mice (6 weeks old) were immunized with proteins *via* subcutaneous injection. A protein dose of 25 µg in a total volume of 200 µL PBS was given with Freund’s Incomplete Adjuvant in 1:1 (vol/vol) emulsion. Secondary immunization was administered 2 weeks later. One week later, blood was collected from mice with their tails severed. After that, serum was extracted from the blood and tested by indirect enzyme-linked immunosorbent assay. Western blots were performed to detect changes in QseB and CapD levels in extracts from wild-type, mutants of *G. parasuis* and Epi conditions. A total of 10 µg protein, as determined by the bicinchoninic acid assay (Biosharp, Hefei, Anhui, China), was loaded per lane and proteins were separated on 12% sodium dodecyl sulfate-polyacrylamide gel electrophoresis. Proteins were then transferred to the polyvinylidene difluoride membrane. After transfer, the polyvinylidene difluoride membrane was incubated with anti-QseB or anti-CapD antiserum at 4°C for 12 h, and Anti-GAPDH Mouse Monoclonal Antibody was from Proteintech Group (Wuhan, Hubei, China). Horseradish peroxidase (HRP) Goat Anti-Mouse IgG (H + L) (Abclonal, Wuhan, Hubei, China) was used as a secondary antibody. The results were visualized with Clarity Western ECL Substrate (Bio-Rad, Hercules, CA, USA).

### Statistical analyses

The comparison of several series was completed using one-way analysis of variance (ANOVA) or two-way ANOVA. Student’s *t*-test was used to determine the differences between groups. Value *P* < 0.05 was regarded as statistically significant.
